# Detection of EGFR mutations with mutation-specific antibodies in stage IV non-small-cell lung cancer

**DOI:** 10.1186/1479-5876-8-135

**Published:** 2010-12-18

**Authors:** Sara Simonetti, Miguel Angel Molina, Cristina Queralt, Itziar de Aguirre, Clara Mayo, Jordi Bertran-Alamillo, José Javier Sanchez, Jose Luis Gonzalez-Larriba, Ulpiano Jimenez, Dolores Isla, Teresa Moran, Santiago Viteri, Carlos Camps, Rosario Garcia-Campelo, Bartomeu Massuti, Susana Benlloch, Santiago Ramon y Cajal, Miquel Taron, Rafael Rosell

**Affiliations:** 1 Pangaea Biotech, USP Dexeus University Institute, Barcelona, Spain; 2 Catalan Institute of Oncology, Hospital Germans Trias i Pujol, Badalona, Barcelona, Spain; 3 Autonomous University of Madrid, Madrid, Spain; 4 Hospital San Carlos, Madrid, Spain; 5 Hospital La Princesa, Madrid, Spain; 6 Hospital Lozano Blesa, Zaragoza, Spain; 7 Hospital General de Valencia, Valencia, Spain; 8 Hospital Juan Canalejo, La Coruña, Spain; 9 Hospital General de Alicante, Alicante, Spain; 10 Hospital Vall d'Hebron, Barcelona, Spain

## Abstract

**Background:**

Immunohistochemistry (IHC) with mutation-specific antibodies may be an ancillary method of detecting EGFR mutations in lung cancer patients.

**Methods:**

EGFR mutation status was analyzed by DNA assays, and compared with IHC results in five non-small-cell lung cancer (NSCLC) cell lines and tumor samples from 78 stage IV NSCLC patients.

**Results:**

IHC correctly identified del 19 in the H1650 and PC9 cell lines, L858R in H1975, and wild-type EGFR in H460 and A549, as well as wild-type EGFR in tumor samples from 22 patients. IHC with the mAb against EGFR with del 19 was highly positive for the protein in all 17 patients with a 15-bp (ELREA) deletion in exon 19, whereas in patients with other deletions, IHC was weakly positive in 3 cases and negative in 9 cases. IHC with the mAb against the L858R mutation showed high positivity for the protein in 25/27 (93%) patients with exon 21 EGFR mutations (all with L858R) but did not identify the L861Q mutation in the remaining two patients.

**Conclusions:**

IHC with mutation-specific mAbs against EGFR is a promising method for detecting EGFR mutations in NSCLC patients. However these mAbs should be validated with additional studies to clarify their possible role in routine clinical practice for screening EGFR mutations in NSCLC patients.

## Background

Non-small-cell lung cancer (NSCLC) is one of the most frequent human malignancies, constituting about 80% of all lung tumors. NSCLC can be divided into genetic subsets on the basis of the activating mutations that they harbor; each of these subsets may correspond to patient cohorts that are likely to benefit from treatment with specific inhibitors[1].

Activating mutations in the epidermal growth factor receptor (EGFR), affecting hotspots within exons that code for the tyrosine kinase domain, can be found in 10-40% of NSCLC patients, mostly in adenocarcinomas, with the higher frequency observed in Asian patients[1,2]. About 50% of mutated patients harbor in-frame deletions in exon 19, (around codons 746 to 750) and 35-45% show the substitution of leucine 858 by an arginine in the exon 21. The remaining mutants are insertions in exon 20 (5%) and uncommon substitutions spanning exons from 18 to 21, such as L861Q[3,4].

These specific mutations are related to a higher sensitivity to the tyrosine kinase inhibitors (TKIs) erlotinib and gefitinib[4-7], whereas the EGFR T790 M mutation in exon 20 is observed in 50% of cases with acquired resistance to erlotinib and gefitinib[8] and has also been detected in 38% of patients with *de novo *drug resistance[9].

Molecular biology techniques, such as SARMS or direct automatic sequencing, are currently used to detect EGFR mutations in formalin-fixed, paraffin-embedded tissues (FFPET). In our experience, in-frame deletions in exon 19 are detected by fragment analysis of fluorescently labeled PCR products, and L858R mutations in exon 21 by TaqMan assay. Mutations are then confirmed by direct sequencing[10,11]. However, the routine use of these methods in clinical laboratories is still often limited by financial and technical constraints. Moreover, their sensitivity depends on the quality and the quantity of tumoral cells in FFPET. In a previous study, we developed a highly sensitive molecular method for detecting EGFR mutations in NSCLC samples containing as few as eight tumor cells[10].

The development of antibodies that specifically detect mutant EGFR protein by IHC would be an easy pre-screening test to complement the molecular assays currently used for the assessment of EGFR mutations in NSCLC. Yu et al[12] have developed mutation-specific rabbit monoclonal antibodies (mAb) against EGFR with the E746_A750 deletion in exon 19 or the L858R point mutation in exon 21 for IHC application (Cell Signaling Technology Inc., Danvers, MA, USA).

In the present study, these two rabbit mAbs were used to assess EGFR mutations in five NSCLC cell lines and in tumor biopsies from 78 stage IV NSCLC patients. The results were then compared with those obtained by other molecular analyses[10,11].

## Methods

### Sources of cell lines and culture

The PC-9 lung tumor cell line was kindly provided by Roche (Basel, Switzerland); the A549 and H460 cell lines were purchased from the American Type Culture Collection. Tissue culture materials were obtained from Biological Industries (Kibbutz Beit Haemek, Israel) and Invitrogen (Paisley, Scotland, UK). H1650 and H1975 were kindly provided by Dr. Herbert Haack and Dr. Katherine Crosby (Cell Signaling Technology, Inc.). We received five slides of the H1975 cell line and five of the H1650 cell line with 4-μm sections for IHC analysis from the Cell Signaling Technology laboratory.

### Study population and tumor pathology

Twenty-six stage IV NSCLC patients had been seen at the USP Dexeus University Institute, and 52 had been previously screened for EGFR mutations and treated with erlotinib as part of the Spanish Lung Adenocarcinoma Data Base (SLADB)[11]. All of these 52 patients were known to have EGFR mutations, while the remaining 26 patients had not been previously screened. All patients provided written informed consent. Approval was obtained from the institutional review board and the ethics committee at each hospital. Table 1 shows patient characteristics.

**Table 1:** Clinicopathological features of the patients analyzed for EGFR mutations by IHC assay.

	Patients (N = 78)	
**Characteristic**	**No**.	**%**

**Age, years**		

Mean	64	
Range	36-85	

**Sex**		

Male	28	36
Female	50	64

**Race**		

Caucasian	78	100

**Smoker**		

Ex-smoker	26	33
Current smoker	7	9
Never smoker	45	58

**Histology**		

Adenocarcinoma	69	88.4
Large-cell carcinoma	5	7.1
Squamous cell carcinoma	1	1.4
Others	3	4.3

**Adenocarcinoma subtype**		

Glandular	36	52.2
Solid	20	29
Papillary	6	8.7
Micropapillary	1	1.4
BAC	6	8.7

Four-μm sections of the FFPET specimens were stained with H/E and histologically examined. All samples were classified according to the 2004 WHO classification[13]: 5 undifferentiated large cell carcinomas and 3 small cell neuroendocrine carcinomas, 1 squamous cell carcinoma and 69 adenocarcinomas, of which 55 showed a single pattern and 14 presented mixed aspects. We further evaluated the adenocarcinoma subtype as follow: 36 adenocarcinomas with a glandular pattern, 20 with a solid aspect, 6 with a partial papillary differentiation, 1 with micropapillary aspects and 6 with a partial bronchioloalveolar pattern (Table 1).

### DNA extraction and mutation analyses

Tumor cells (8 to 150) were captured by laser microdissection (Carl Zeiss MicroImaging GmbH, München, Germany) into 10 μL of PCR buffer (Ecogen, Barcelona, Spain) plus proteinase K and incubated 4 hours to overnight at 60°C. Proteinase was inactivated at 95°C for 10 min, and the cell extract submitted to PCR. DNA from the cell line PC-9 was used as a mutated control for exon 19, and wt control for exons 20 and 21. DNA from the H1975 cell line was used as a wt control for exon 19, and mutated control for exons 21/20.

EGFR gene mutations in exons 19 and 21 were analyzed by our sensitive methodology as previously described[10]. Exons 19 and 21 of the EGFR gene were amplified by a nested PCR. Sequencing was performed using forward and reverse nested primers with the ABI Prism 3100 DNA Analyzer (Applied Biosystems, Foster City, CA, USA). In addition to sequencing, EGFR deletions in exon 19 were determined by length analysis of fluorescently labeled PCR products. The collected data were evaluated with the GeneScan Analysis Software (Applera, Norwalk, CT, USA). Finally, EGFR mutation (L858R) in exon 21 was also determined by TaqMan^® ^Assay (Applied Biosystems). The L861Q mutation was detected by direct sequencing.

### Immunohistochemical analysis

The following antibodies were used for the IHC analysis (Cell Signaling Technology, Inc.): EGF Receptor (D38B1), EGFR E746-A750 deletion specific (6B6) and EGFR L858R mutant-specific (43B2). The FFPET samples were cut serially at 4 μm and the sections were introduced in the stainer and automatically deparaffinized (Leica Microsystems BondMAX Automated Immunostainer, Wetzlar, Germany). The reactives were added automatically, treating the samples with EDTA buffer (pH 9.0) (Bond Epitope Retrieval Solution 2, Leica Microsystems) as antigen retriever and processed for 30 min. The slides were incubated with the antibodies against EGF receptor and EGFR mutations at a dilution of 1:100 for 60 minutes. After the sections were treated with the streptavidin-biotin-peroxidase complex method (Bond Polymer Refine Detection, Leica Microsystems) with diaminobenzidine (DAB) as a chromogen and counterstained with hematoxylin.

IHC expression of mAbs against EGFR was evaluated using the following scoring, as previously described[14]: 0 = negative or faint staining in <10% of tumor cells; 1 = weak staining in >10% of cancer cells; 2 = moderate staining; 3 = strong staining. A score of 0 was considered negative, a score of 1 was considered weakly positive, and a score of 2 or 3 was considered highly positive (Additional File 1, Figure S1).

### Statistical analyses

The absolute and relative frequencies of qualitative variables were calculated in percentages. The sensitivity and specificity of the EGFR test by IHC was determined in comparison with PCR-based results. All analyses were performed using SPSS v 16.0 software (SPSS Inc., Chicago, IL).

## Results

### EGFR mutation analysis in NSCLC patients

We screened EGFR mutations in 78 FFPET samples from NSCLC patients by a methodology described elsewhere[10], which involves fragment analysis (exon 19), Taqman assay (exon 21) and sequencing. Twenty-six samples were analyzed in the Pangaea Biotech Oncology Laboratory and 52 from a previous study[11] were analyzed in the Catalan Institute of Oncology, Hospital Germans Trias i Pujol. Twenty-two samples (28%) were wt EGFR, 29 (37%) had a deletion in exon 19, and 27 (35%) had mutations in exon 21. Of the 29 patients with the exon 19 deletion, 17 (59%) had 15-bp deletions (16 with del E746-A750 [ELREA] and 1 with del E746-A750 [ELREA] + T751I), and 12 (41%) had rare deletions of 9-bp, 12-bp, 18-bp, 21-bp or 24-bp. Of the 27 patients with exon 21 mutations, 25 (93%) had the L858R mutation and 2 (7%) had the L861Q mutation (Additional File 1, Table S1).

### IHC analysis of mutation-specific mAbs against EGFR in human NSCLC cell lines

We analyzed by IHC five human NSCLC cell lines with known EGFR gene status. In the two cell lines with wt EGFR (H460 and A549), we found positive (score 3) expression of EGFR (D38B1) protein (100%) and negative (score 0) expression of EGFR E746-A750 deletion specific (6B6) and EGFR L858R mutant-specific (43B2).

In the two cell lines with exon 19 deletion (15 bp) (H1650 and PC9), expression of EGFR (D38B1) protein and EGFR E746-A750 deletion specific (6B6) was positive (score 3) (100%) and expression of EGFR L858R mutant-specific (43B2) was negative (score 0).

**Figure 1:**
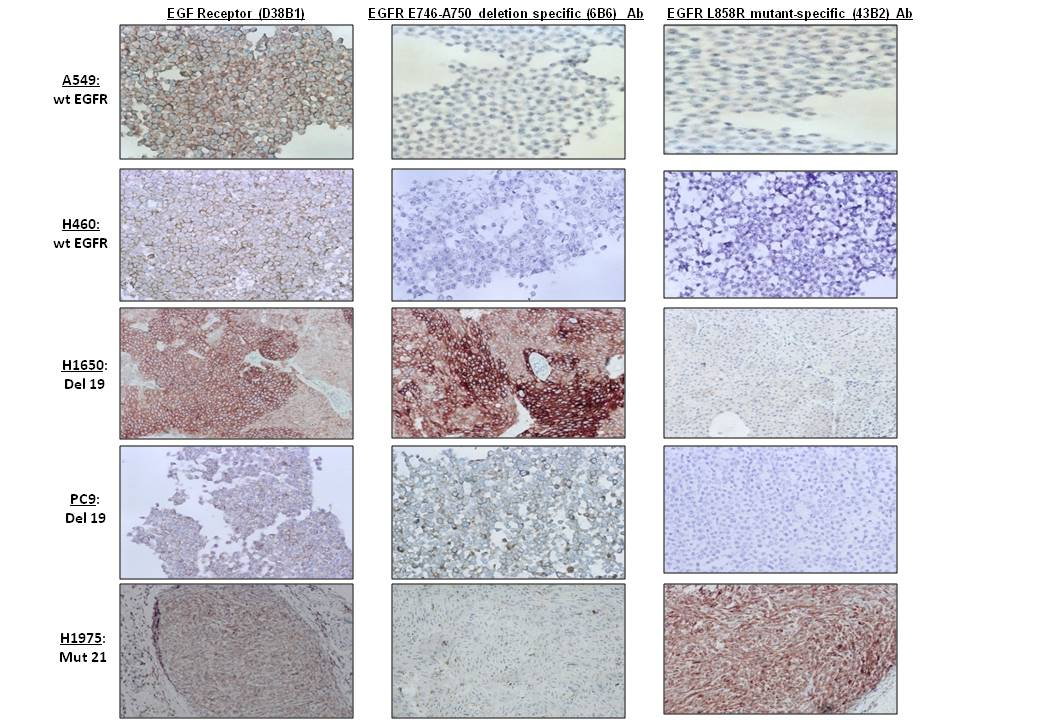
**IHC analysis of EGFR mutations in five human NSCLC cell lines**. A549 and H460 showed negativity for both mutation-specific antibodies. EGFR E746-A750 deletion specific (6B6) antibody stained H1650 and PC9 harboring the exon 19 deletion, and EGFR L858R mutant-specific (43B2) antibody stained the H1975 cell line with exon 21 mutation.

The cell line H1975 with exon 21 mutation (L858R) showed positivity (score 3) for EGFR (D38B1) and EGFR L858R mutant-specific (43B2) (100%) and negativity (score 0) for EGFR E746-A750 deletion specific (6B6). (Table 2, Figure 1).

**Table 2:** IHC expression of EGFR mutation antibodies in human NSCLC cell lines and in NSCLC tumor tissues

EGFR mutation status	EGFR (D38B1) Ab (+)	EGFR E746-A750 deletion-specific (6B6) Ab (+)	EGFR L858R mutant-specific (43B2) Ab (+)
**H460 and A549 (WT)**	2/2 (100%)	0/2 (0%)	0/2 (0%)

**H1650 and PC9 (DEL 19)**	2/2 (100%)	2/2 (100%)	0/2 (0%)

**H1975 (L858R + T790M)**	1/1 (100%)	0/1 (0%)	1/1 (100%)

			

**Tumor Tissue (WT; N = 22)**	8/22 (36%)	0/22 (0%)	0/22(0%)

**Tumor Tissue (DEL 19;N = 29)**	28/29 (97%)	20/29 (69%)	0/29 (0%)

**Tumor Tissue (MUT 21;N = 27)**	26/27 (96%)	0/27 (0%)	25/27 (93%)

Abbreviations: WT: wild-type. DEL 19: Exon 19 deletion; MUT 21: Exon 21 mutation.

### IHC analysis of mutation-specific mAbs against EGFR in NSCLC patients

In 22 tumor tissues with wt EGFR, we found high expression of EGFR (D38B1) protein (score 2 or 3) in 8 cases (36%) and weak positivity (score 1) in 4 cases (18%). All the cases were negative for EGFR E746-A750 deletion specific (6B6) and EGFR L858R mutant-specific (43B2) proteins.

In the 29 patients with exon 19 deletions, high expression of EGFR E746-A750 deletion-specific (6B6) protein (score 2 or 3) was observed in 17/17 cases (100%) with 15-bp deletion (16 with ELREA and 1 with ELREA + T751I). Of the 12 cases showing uncommon deletions in exon 19, nine (75%) samples were completely negative (score 0) and 3 (25%) were weakly positive (score 1). All cases were negative (score 0) for EGFR L858R mutant-specific (43B2) protein.

**Figure 2:**
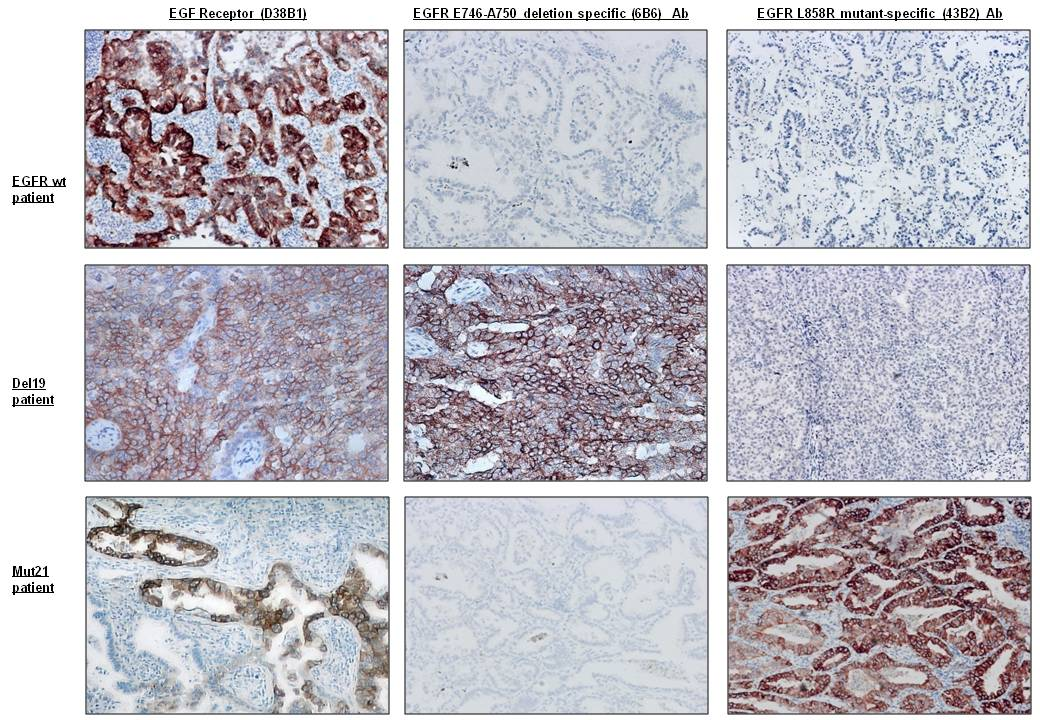
**IHC staining of tumor samples from lung cancer patients**. EGFR E746-A750 deletion specific (6B6) antibody detected 100% of cases with the 15-bp exon 19 deletion, and EGFR L858R mutant-specific (43B2) antibody detected 100% of cases harboring L858R mutation of exon 21.

**Figure 3:**
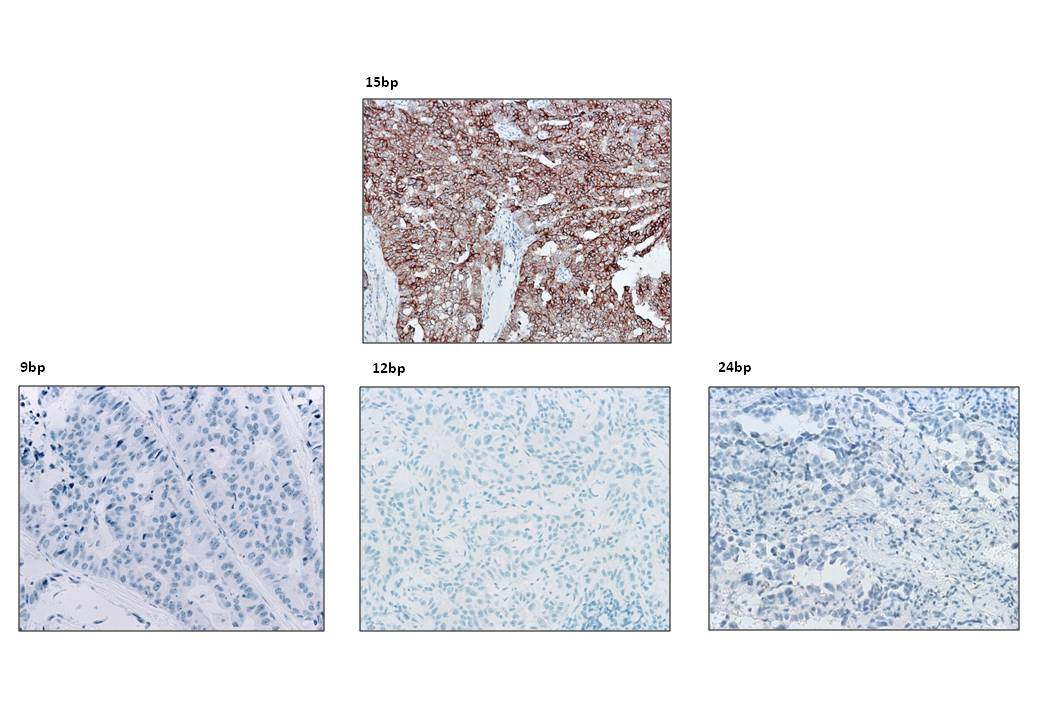
**Expression of EGFR E746-A750 deletion specific (6B6) protein in the different types of exon 19 deletions**. Among samples (12/29) showing negative or weak protein expression (score 0 or 1) 4 cases had a 9-bp deletion, 1 case had a 12-bp deletion, 5 cases had 18-bp deletion, 1 case had 21-bp deletion, and 1 case had a 24-bp deletion.

In the 27 patients with exon 21 mutations, IHC with the mAb against the L858R mutation showed high positivity for the protein (score 2 or 3) in 25/27 (93%) and in 100% of the 25 samples with the L858R substitution; however, it failed to identify the L816Q mutation (0/2 cases). In addition, all 27 samples were negative for the EGFR E746-A750 deletion-specific (6B6) protein (Tables 2 and 3, Figures 2 and 3).

**Table 3:** Correlation of IHC expression of mutation-specific antibodies and EGFR exon 19 deletion subtype analyzed by GeneScan, TaqMan and direct sequencing.

EGFR EXON 19 DELETION SUBTYPE	0	1+	2+	3+
**15 bp** **N = 17**	0/17 (0%)	0/17 (0%)	2/17 (11%)	15/17 (89%)

**9 bp** **N = 4**	2/4 (50%)	2/4(50%)	0/4 (0%)	0/4 (0%)

**12 bp** **N = 1**	1/1 (100%)	0/1 (0%)	0/1 (0%)	0/1 (0%)

**18 bp** **N = 5**	4/5 (80%)	1/5 (20%)	0/5 (0%)	0/5 (0%)

**21 bp** **N = 1**	1/1 (100%)	0/1 (0%)	0/1 (0%)	0/1 (0%)

**24 bp** **N = 1**	1/1 (100%)	0/1 (0%)	0/1 (0%)	0/1 (0%)

## Discussion

EGFR is a member of the ErbB family of receptor tyrosine kinases, which also includes HER2/neu, HER3, and HER4[15]. Activating mutations in the tyrosine kinase domain, involving mainly exons 19 and 21, play an important role in lung oncogenesis and tumor progression and are related to the clinical efficacy of EGFR TKIs such as gefitinib or erlotinib[5,9,11]. Analysis of these mutations has become an important tool for targeted therapy in lung cancer^3^, and in recent years many efforts have been made to find a more specific and sensitive methodology to detect them[10,16-18]. Nevertheless, these techniques are relatively expensive for routine use in clinical laboratories, and depend on the quality of the samples. IHC is a standardized assay of simple methodology and high sensitivity and specificity, and the development of specific antibodies against EGFR mutation proteins might be useful for the diagnosis and treatment of lung cancer.

In 2009 Yu et al [12] first generated two mAbs against E746-A750del and L858R point mutation from New Zealand rabbits and evaluated them by Western blotting, immunofluorescence and IHC. They tested these antibodies in a series of cell lines and in tumor tissues from patients with primary NSCLC, with known and unknown EGFR mutations, comparing the IHC results with DNA sequencing. They found that IHC with these mutation-specific antibodies for EGFR mutations showed a sensitivity of 92% and a specificity of 99%.

Recently, five studies[14,19-22] examined the presence of EGFR mutations in NSCLC by IHC using the same two rabbit mAbs and reported sensitivity ranging from 36% to 100% and specificity ranging from 94% to 99% (Table 4). Kato et al [20] analyzed 70 gefitinib-treated NSCLC patients. Although a high sensitivity and specificity for these mAbs were described, IHC staining was not significantly correlated with overall survival. A very exhaustive analysis of the role of EGFR in NSCLC was recently reported by Ilie et al [19]. They assessed EGFR status in a tissue microarray (TMA) of 61 lung adenocarcinomas by IHC, fluorescent in situ hybridization (FISH) and direct sequencing and compared their results with those of conventional methods performed on whole-tissue sections. The authors reported a specificity of 92% for the mAb against the E746-750 deletion. Kawahara et al [21] reported a sensitivity of 83% for the L858R mutation antibody and 79% for the exon 19 deletion antibody. Brevet et al [14] reported a sensitivity of 84.6% and a specificity of 98.9% for E746-A750 and a sensitivity of 95.2% and a specificity of 98.8% for L858R. Kitamura et al [22] reported a sensitivity of 36% and a specificity of 97% for L858R and a sensitivity of 40% and a specificity of 99% for E746-A750. In the present study, we found a sensitivity of 100% and a specificity of 100% for the L858R exon 21 mutation antibody and a sensitivity of 63% and a specificity of 100% for the 15-bp deletion antibody. Table 4 summarizes the clinicopathological characteristics of patients and the findings of seven studies examining EGFR mutations by IHC, including the present study.

**Table 4:** Patient characteristics and EGFR mutation status in seven studies examining EGFR mutations by IHC. (Blank cells indicate that information is not available.)

	Simonetti et al	**Ilie et al **[19]	**Kato et al **[20]	**Kitamura et al **[22]	**Brevet et al **[14]	**Yu et al **[12]	**Kawahara et al **[21]
**Total cases**	78	61	70	343	194	340	60

**Age, years**							

range	36-85	42-83	27-88				

median	64	67	59.9				

**Sex**							

male	28	31	36				

female	50	30	34				

**Ethnicity**							

Caucasian	78	61	0	0			0

Asian	0	0	70	343			60

**Smoking history**							

smokers	33	37	41				

non-smokers	45	24	29				

**Histology**							

adeno	69	61	57			217	60

SCC	1	0	7			112	0

LCC	5	0	4			11	0

others	3	0	2			0	0

**EGFR**							

exon 19	29	10	18	21	55	58	21

exon21	27	0	12	14	18	56	23

wild-type	22	51	29	296	145	167	16

**IHC sensitivity**							

overall						92%	

delE746-A750 Ab	63%	22.86%	81.1%	99%	84.6%		79%

l858r Ab	100%		75%	97%	95.2%		83%

**IHC specificity**							

overall						99%	

delE746-A750 Ab	100%	92%	100%	40%	98.8%		

L858R Ab	100%		96.6%	36%	98.8%		

Although the most common EGFR mutations are the 15-bp ELREA deletion in exon 19 and the L858R substitution in exon 21[2,3], other less frequent deletions have been identified[4,6,8,23]. Using DNA sequencing, Yu et al [12] detected only two cases with uncommon deletions in exon 19; E746-T751 del stained positive and L747-A750 negative for IHC. In the present study, we had 12 samples with uncommon deletions in exon 19 (9-bp, 12-bp, 18-bp, 21-bp and 24-bp) and 2 samples with the uncommon exon 21 L816Q mutation. In these samples, IHC for both mutation-specific antibodies was not able to detect the alteration.

## Conclusions

IHC with the mutation-specific rabbit mAbs against EGFR is a simple and standardized assay which could prove useful as a first, quick screening of NSCLC patients. However, although these antibodies seem to be quite reliable for the detection of patients carrying the most common EGFR mutations[12], they were not able to detect other EGFR gene mutations, such as 9-bp, 12-bp, 18-bp, 21-bp or 24-bp deletions or the L861Q substitution[14]. In consequence, if the antibodies are to be used in clinical practice, molecular biology techniques will be needed to further analyze the IHC-negative patients. However, the generation of a refined panel of antibodies able to detect both the frequent and the uncommon EGFR exon 19 deletions and exon 21 mutations as well as the resistance mutation T790 M in exon 20 could lead to the universal application of IHC for detecting EGFR mutations in NSCLC patients, as part of the routine IHC work-up of lung adenocarcinomas.

## Competing interests

The authors declare that they have no competing interests.

## Authors' contributions

SS, MT, RR participated in the design of the study and its writing. MAM, CQ, IDA, CM, JBA, SB carried out the molecular genetic studies. JLGL, UJ, DI, TM, SV, CC, RGC, BM have made substantial contributions to acquisition of data. JJS, MT, RR, SS have made substantial contributions to analysis and interpretation of data. SS, SRC carried out the immunoassays. JJS performed the statistical analysis. All authors read and approved the final manuscript.

## Supplementary Material

Additional file 1 **Table S1**. Table showing EGFR mutation status as detected by our sensitive methodology. **Figure S1**. Images showing scoring of IHC staining of human NSCLC cell lines and lung cancer patient tumor tissues. A score of 0 was considered negative, a score of 1 was considered weakly positive, and a score of 2 or 3 was considered strongly positive Click here for file
