# Supplementary material for: Detection of EGFR mutations with mutation-specific antibodies in stage IV non-small-cell lung cancer
Source: J Transl Med. 2010 Dec 18;8:135. doi: 10.1186/1479-5876-8-135 (PMC3016260; doi:10.1186/1479-5876-8-135)

**ADDITIONAL FILE 1**

**Table S1.** EGFR mutation status detected by our sensitive methodology.

| **Cases** | **Histologic Subtype** | **Adenocarcinoma Subtype** | **Exon 19 mutation position** |
| --- | --- | --- | --- |
| **H460** | UNDIFFERENTIATED LARGE CELL CARCINOMA |  | wt |
| **A549** | LUNG ADENOCARCINOMA | **BAC** | wt |
| **H1650** | NSCLC |  | del E746-A750 (ELREA) |
| **PC9** | LUNG ADENOCARCINOMA |  | del E746-A750 (ELREA) |
| **H1975** | LUNG ADENOCARCINOMA |  | L858R |
| **1** | LUNG ADENOCARCINOMA | glandular | wt |
| **2** | LUNG ADENOCARCINOMA | glandular | wt |
| **3** | LUNG ADENOCARCINOMA | glandular + **BAC** | wt |
| **4** | LUNG ADENOCARCINOMA | solid | wt |
| **5** | LUNG ADENOCARCINOMA | glandular | wt |
| **6** | LUNG ADENOCARCINOMA | glandular | wt |
| **7** | LUNG ADENOCARCINOMA | glandular | wt |
| **8** | LUNG ADENOCARCINOMA | glandular | wt |
| **9** | SMALL CELL LUNG CARCINOMA |  | wt |
| **10** | LUNG ADENOCARCINOMA | papillary + micropapillary | wt |
| **11** | LUNG ADENOCARCINOMA | glandular | wt |
| **12** | SMALL CELL LUNG CARCINOMA |  | wt |
| **13** | LUNG ADENOCARCINOMA | glandular + papillary | wt |
| **14** | LUNG ADENOCARCINOMA | glandular | wt |
| **15** | SMALL CELL LUNG CARCINOMA |  | wt |
| **16** | UNDIFFERENTIATED LARGE CELL CARCINOMA |  | wt |
| **17** | UNDIFFERENTIATED LARGE CELL CARCINOMA |  | wt |
| **18** | UNDIFFERENTIATED LARGE CELL CARCINOMA |  | wt |
| **19** | LUNG ADENOCARCINOMA | glandular | wt |
| **20** | UNDIFFERENTIATED LARGE CELL CARCINOMA |  | wt |
| **21** | SQUAMOUS CELL CARCINOMA |  | wt |
| **22** | LUNG ADENOCARCINOMA | glandular | wt |
| **23** | LUNG ADENOCARCINOMA | papillary + **BAC** | del T751-E758 +ins A |
| **24** | LUNG ADENOCARCINOMA | glandular | del E746-A750 |
| **25** | LUNG ADENOCARCINOMA | glandular | del E746-A750 |
| **26** | LUNG ADENOCARCINOMA | solid | del E746-A750 |
| **27** | LUNG ADENOCARCINOMA | solid | del E746-A750 |
| **28** | LUNG ADENOCARCINOMA | glandular + solid | del E746-A750 + T751I |
| **29** | LUNG ADENOCARCINOMA | glandular | del E746-A750 |
| **30** | LUNG ADENOCARCINOMA | glandular | del E746-A750 |
| **31** | LUNG ADENOCARCINOMA | solid + glandular | del E746-A750 |
| **32** | LUNG ADENOCARCINOMA | papillary | del E746-A750 |
| **33** | LUNG ADENOCARCINOMA | glandular | del E746-A750 |
| **34** | LUNG ADENOCARCINOMA | glandular | del L747-E749 |
| **35** | LUNG ADENOCARCINOMA | glandular + **BAC** | del E746-E749 |
| **36** | LUNG ADENOCARCINOMA | papillary | del E746-A750 |
| **37** | LUNG ADENOCARCINOMA | glandular | del E746-A750 |
| **38** | UNDIFFERENTIATED LARGE CELL CARCINOMA |  | del E747-E749 + ins C |
| **39** | LUNG ADENOCARCINOMA | glandular | del E746-A750 |
| **40** | LUNG ADENOCARCINOMA | solid | del E746-A750 |
| **41** | LUNG ADENOCARCINOMA | glandular | del E746-A750 |
| **42** | LUNG ADENOCARCINOMA | solid | del E746-A750 |
| **43** | LUNG ADENOCARCINOMA | solid + papillary | del E746-A750 |
| **44** | LUNG ADENOCARCINOMA | glandular | L858R |
| **45** | LUNG ADENOCARCINOMA | solid | L858R |
| **46** | LUNG ADENOCARCINOMA | papillary + **BAC** | L858R |
| **47** | LUNG ADENOCARCINOMA | papillary + **BAC** | L858R |
| **48** | LUNG ADENOCARCINOMA | solid | L858R |
| **49** | LUNG ADENOCARCINOMA | solid | L858R |
| **50** | LUNG ADENOCARCINOMA | glandular | L858R |
| **51** | LUNG ADENOCARCINOMA | papillary | L858R |
| **52** | LUNG ADENOCARCINOMA | solid | L858R |
| **53** | LUNG ADENOCARCINOMA | glandular | L858R |
| **54** | LUNG ADENOCARCINOMA | glandular | L858R |
| **55** | LUNG ADENOCARCINOMA | solid | L858R |
| **56** | LUNG ADENOCARCINOMA | glandular | L861Q |
| **57** | LUNG ADENOCARCINOMA | solid | L858R |
| **58** | LUNG ADENOCARCINOMA | solid | L858R |
| **59** | LUNG ADENOCARCINOMA | glandular + **BAC** | L858R |
| **60** | LUNG ADENOCARCINOMA | solid | L861Q |
| **61** | LUNG ADENOCARCINOMA | glandular | L858R |
| **62** | LUNG ADENOCARCINOMA | papillary | L858R |
| **63** | LUNG ADENOCARCINOMA | micropapillary | L858R |
| **64** | LUNG ADENOCARCINOMA | solid | L858R |
| **65** | LUNG ADENOCARCINOMA | glandular | L858R |
| **66** | LUNG ADENOCARCINOMA | solid | L858R |
| **67** | LUNG ADENOCARCINOMA | glandular | L858R |
| **68** | LUNG ADENOCARCINOMA | glandular + papillary | L858R |
| **69** | LUNG ADENOCARCINOMA | glandular + papillary + solid | L858R |
| **70** | LUNG ADENOCARCINOMA | glandular | L858R |
| **71** | LUNG ADENOCARCINOMA | glandular | del L747-E749 |
| **72** | LUNG ADENOCARCINOMA | glandular | del L747-E749 |
| **73** | LUNG ADENOCARCINOMA | glandular | del L747-753 |
| **74** | LUNG ADENOCARCINOMA | glandular + micropapillary | del L747-753 |
| **75** | LUNG ADENOCARCINOMA | papillary | del E746-753 |
| **76** | LUNG ADENOCARCINOMA | solid | del L747-753 |
| **77** | LUNG ADENOCARCINOMA | solid | del E746-753 |
| **78** | LUNG ADENOCARCINOMA | solid | del L747-753 + ins C+G |

Abbreviations: BAC, Bronchioloalveolar adenocarcinoma. NSCLC, Non-small cell lung cancer.

**Figure S1.** Scoring of IHC staining of human NSCLC cell lines and lung cancer patient tumor tissues. A score of 0 was considered negative, a score of 1 was considered weakly positive, and a score of 2 or 3 was considered strongly positive.


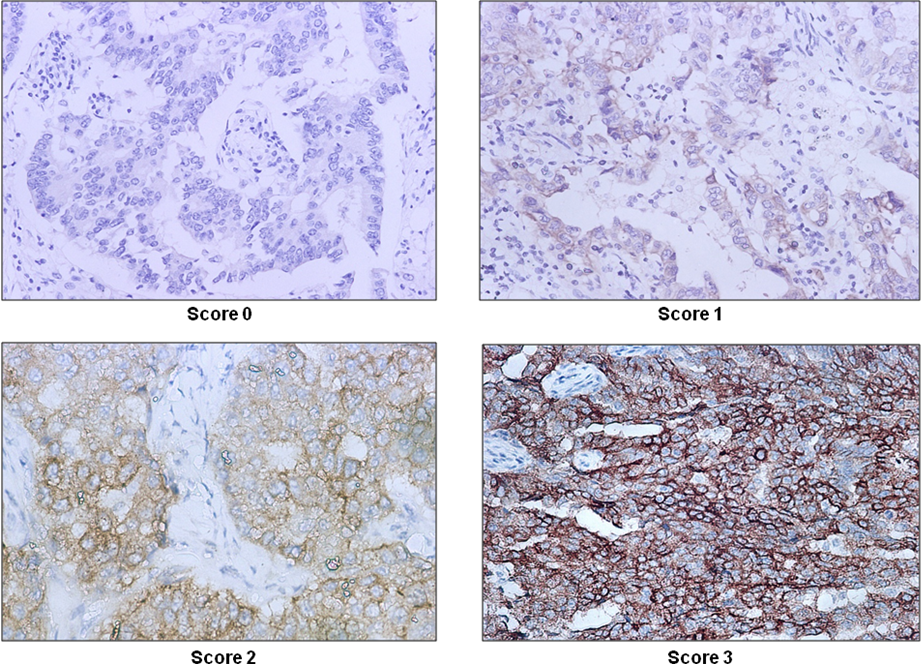

Supplement: Additional file 1 — Table S1. Table showing EGFR mutation status as detected by our sensitive methodology. Figure S1. Images showing scoring of IHC staining of human NSCLC cell lines and lung cancer patient tumor tissues. A score of 0 was considered negative, a score of 1 was considered weakly positive, and a score of 2 or 3 was considered strongly positive [file 1479-5876-8-135-S1.DOC]
